# Automated detection of patterned single-cells within hydrogel using deep learning

**DOI:** 10.1038/s41598-022-22774-0

**Published:** 2022-10-31

**Authors:** Tanmay Debnath, Ren Hattori, Shunya Okamoto, Takayuki Shibata, Tuhin Subhra Santra, Moeto Nagai

**Affiliations:** 1 Department of Mechanical Engineering, Toyohashi University of Technology, Toyohashi, Aichi 441-8580 Japan; 2 Indian Institute of Technology Madras, Chennai, Tamil Nadu 600036 India; 3 Electronic Inspired Interdisciplinary Research Institute (EIIRIS), Toyohashi University of Technology, Toyohashi, Aichi 441-8580 Japan

**Keywords:** Data acquisition, Machine learning, Biomedical engineering

## Abstract

Single-cell analysis has been widely used in various biomedical engineering applications, ranging from cancer diagnostics, and immune response monitoring to drug screening. Single-cell isolation is fundamental for observing single-cell activities and an automatic finding method of accurate and reliable cell detection with few possible human errors is also essential. This paper reports trapping single cells into photo patternable hydrogel microwell arrays and isolating them. Additionally, we present an object detection-based DL algorithm that detects single cells in microwell arrays and predicts the presence of cells in resource-limited environments at the highest possible mAP (mean average precision) of 0.989 with an average inference time of 0.06 s. This algorithm leads to the enhancement of the high-throughput single-cell analysis, establishing high detection precision and reduced experimentation time.

## Introduction

A cell is the most basic unit of life^1^ and studying cellular structures, functions, and properties reveals the complex biochemical and physiological phenomena, leading to the understanding of pathological activities^2^. In such a case, a single cell provides accurate information regarding the different physiological and biochemical states and processes^3^. Hence, the research community has invested much attention in the applications of single cells and the development of single-cell assays to detect the characteristics of such cells^4^. Single cells are often counted by human eyes and this human engagement reduces the productivity of data processing.

Over the years, various passive methodologies have been explored, ranging from non-adhesive phospholipids^5^ to photolithography-based techniques^6–8^ and micro-contact printing^9^ that enable cell adhesion and patterning. Moreover, extensive research on pattern longevity and biocompatibility has expanded the utility of such protocols to various long-term bio-applications. Cell patterning was demonstrated by patterning glass with a layer of non-adhesive phospholipids^10^. Patterning was achieved using photo-sensitive materials and through a mask, laminin was patterned by UV light to direct neurite development along unirradiated laminin channels^5^. A breakthrough came when the photolithography process was introduced in this process and a photoresist was applied to a substrate before being exposed to radiation using a photomask. The deposited protein on the photoresist was removed leaving a pattern that resembles the original pattern on the photomask^6^. One of the major advantages of photolithography is that it reaches sub-micron levels and can covalently bond patterned molecules to a substrate to create long-lasting patterns. However, the usage of harsh solvents during the process limits its application to chemical-resistant biomolecules. µCP (Microcontact printing) was also used for cell patterning. Bioactive compounds or SAM (self-assembled monolayer) were deposited to manipulate the property of a 2-D surface^9^. Pattern longevity is a concern in this method because of the increased reliance on adsorption. Photolithography and plasma thin-film polymerization have been used to pattern two-dimensional in vitro cell cultures^7^. They enable the deposition of polymeric films with thicknesses ranging from a few nanometers to micrometers, provided that the plasma conditions are appropriate. Recent advances in cell capturing with hydrogel include the development of pocket-like structures through photolithography. The use of photo-acid-generating polymers in the photolithographic construction of distinctive microstructures made of flexible hydrogel sheets was proposed. Following area-selective micropatterned irradiation, a variety of pocket-like microstructures were created by controlling the lifting off of the crosslinked polymer from the substrate and the crosslinking of a hydroxyl-rich polymer^8^. The authors mentioned that this method is a feasible technology for bio applications.

One of the most extensively studied and used hydrogel is the bioresist, consisting of PNIPAAm (poly-(N-isopropyl acrylamide))^11^, because of its biocompatibility and high photopatternability. Over the years, PNIPAAm and its modified interfaces^12–14^ have been experimented with different cell lines^11–13,15^ to enhance cell adhesion. Some of these extensive studies include the combination of PNIPAAm with biological ligands, which enhanced cell adhesion and proliferation. Firstly, a cell adhesive peptide with an arginine-glycine-aspartic acid-serine (RGDS) was used as a ligand of the thermoresponsive cell culture dish for observing HUVEC cell adhesion and proliferation under serum-free conditions^11^. Later, an insulin-immobilized thermoresponsive cell culture dish was also investigated for cell proliferation. Insulin was used with poly-(NIPAAm-co-CIPAAm) and tested with BAEC cells, resulting in observable cell adhesion^12^. Similarly, bFGF (basic fibroblast growth factor) was immobilized on a thermoresponsive cell culture dish to investigate a mouse fibroblast (NIH/3 T3) proliferation^15^. Moreover, over the years, it has been observed that researchers have experimented with different patterned PNIPAAm-modified interfaces. Researchers mentioned creating surfaces of thermoresponsive cell culture using microcontact printing. Microcontact printing was used to imprint fibronectin in a line pattern on the PNIPAAm-grafted dish. On the dish, SMC cells were seeded and cultured till confluency. By lowering the temperature, an SMC sheet with a certain orientation level was recovered in the culture dish^13^. Similarly, the reversible addition-fragmentation chain transfer (RAFT) polymerization process was used to create a line pattern made up of PNIPAAm, which adheres to cells, and PNIPAAm-b-poly (N-acryloyl morpholine), which does not^14^. On the patterned surface, normal human dermal fibroblasts (NHDFs) adhered, proliferated, and aligned with the line pattern. NHDF cell sheet was successfully obtained by reducing the temperature of the system. The patternability and biocompatibility of bioresist make the compound suitable for biological applications and have also inspired us to explore further in our study.

Cell adhesion and patterning were achieved by a wide-ranging of methods, other than bioresist, ranging from different biocompatible chemical groups^16–18^ to different photopatterning methodologies^16,19^. Initially, surface patterning was also realized photochemically. Chemical micropatterns for cell adhesion have been produced using surface-immobilized photoreactive chemicals. UV or laser activation immobilized benzophenone groups and oligopeptides with the Arg-Gly-Asp (RGD) sequence on top of ethylene glycol modified-alkanethiols^16^. Since the thiols are prone to oxidize in ambient settings, they are probably not suitable for long-term cell culture. Later, a biocompatible photoresist was proposed using NVP (*N*-vinyl-2-pyrrolidone) lactam ring and MMA (methyl methacrylate) as the biocompatible units, with *t*-BOC (*t*-butoxy carbonyl) functional group as the protection group^17^. Fibroblast cells were maintained on these patterned samples, and after two weeks they had collagen deposition and a clearly defined cellular orientation. PAA (Poly acrylic acid) was also explored to control the cell behaviors of different cell lines. Ion beam lithography was used to prepare PAA-patterned polystyrene substrates to influence the behaviors of mouse fibroblasts and human embryonic kidney cells^19^. Poly- or OEGMA (oligo (ethylene glycol) methacrylate) groups have been explored as well. It has been shown that copolymers of MEO_2_MA (2-(2-methoxy ethoxy) ethyl methacrylate) and OMEGA exhibited thermo-responsive behavior which was tuned based on the composition of the corresponding monomers^18^. Cells adhered to the surface of the medium at 37 °C, but when the temperature was dropped to 25 °C, a rounding of the cells was seen, facilitating simple separation from the surface without trypsinization.

Single cells need to be automatically characterized to reduce analysis time and efficiently reveal new findings. With the advancement in DL (deep learning) and artificial intelligence, there has been a development in intelligent tools that can perform image analysis on microscopic data^20,21^ and eventually automate the entire process of data collection and artifact detections^22,23^. Recent advancements in the application of deep learning networks range from graph-based cell detection and score estimation, cell counting^20^, and cell segmentation^21^, to fully developed pipelines that characterize the development of cells under given conditions^22^. The functionalities of DL are not restricted to the physical morphology, rather literature shows that image-based cell phenotyping has also been realized in recent years^24^. Similarly, in new applications of DL that microscopic structures, microfluidic droplets were detected and analyzed using state-of-the-art algorithms^23^. The authors mentioned having experimented with Faster region-based convolutional neural networks (R-CNN)-based techniques for the identification of droplets in microchannels. Essentially, these studies suggest that other cell-related tasks, including cell detection pipelines, can be automated as well and applicable to trapped cells within microwells.

**Figure 1:**
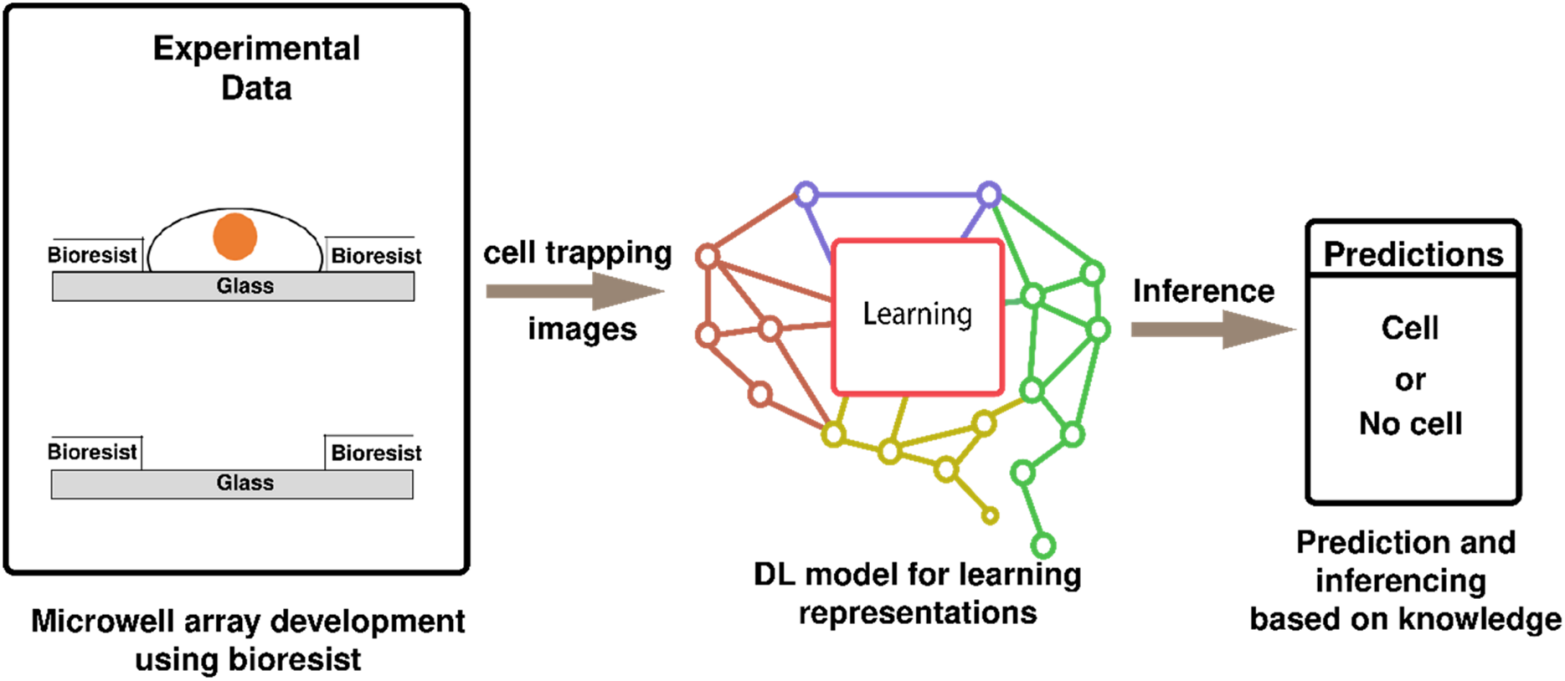
(**A**) schematic overview of the work reported in the paper. In the paper, we have demonstrated a novel method of developing microwell arrays using photo patternable bioresists. This enhances the biocompatibility of the developed microstructures. We captured images after seeding cells in microwells to observe cell trapping. We prepared a DL model that would learn important representations from those images, like microwell location and cell structure, etc., and would be able to detect the presence of single cells within a particular microwell.

In this study, we have introduced a novel method of single-cell trapping using bioresist patterning, where microwell-like structures have been manufactured to trap single cells from the cell suspension. Bioresist was patterned by photolithography, which makes the system convenient and stable. Moreover, this process is highly reproducible. Bioresists are biocompatible in nature and hence can withstand a longer incubation time in the cell medium, facilitating longer cell behavior observation time. Additionally, we compared different object detection models to detect single cells in microwell arrays and classify the presence of cells. We have shown how neural networks when trained on custom microwell array datasets are robust and capable enough to independently detect cells in microwell arrays within a considerably restricted timeframe, under resource-constrained environments. Empirically, we have shown that the models and their training parameters that have been opted for this study outperformed the recent advances in cell detection and characterization, thus suggesting the possible implementation of the DL models for such high-throughput experiments. Figure 1 shows a schematic overview of the entire work that we have reported in this paper.

## Materials and methods

### Preparation of microstructure patterns

**Figure 2:**
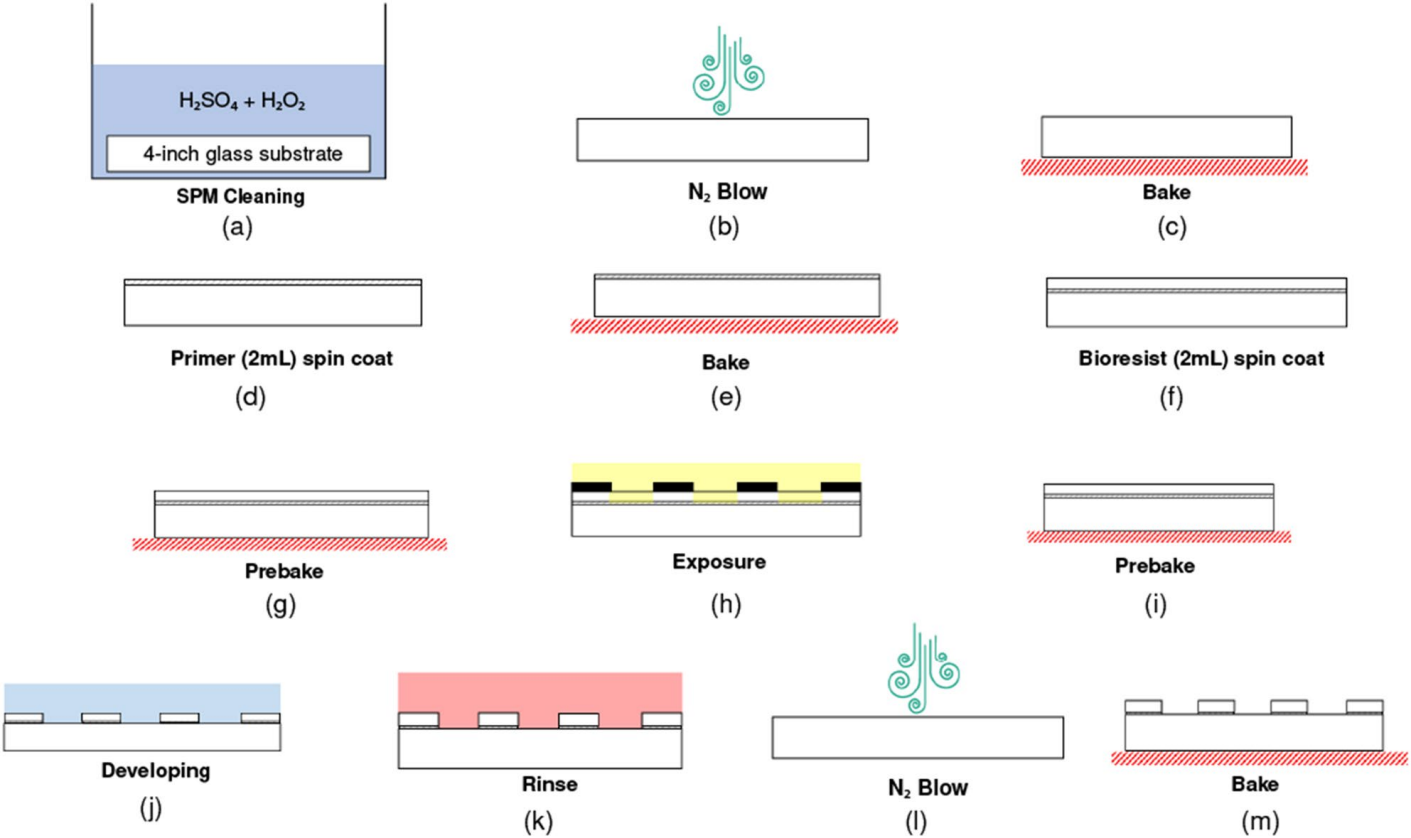
Photolithography process of bioresist hydrogel on a glass substrate. (**a**) The wafer was cleaned in SPM solution (H_2_SO_4_ and H_2_O_2_ solution in 1:1 ratio) for 15 min. (**b**) N_2_ blow. (**c**) The substrate was baked at 180 °C for 10 min and then was brought to room temperature for 5 min. (**d**) Primer was spin-coated with a slope for 5 s; spun at 500 rpm for 20 s; slope for 5 s; spun for 60 s at 3000 rpm; slope for 10 s. (**e**) The substrate was baked in the following configurations: 65 °C for 2 min; 120 °C for 10 min; 65 °C for 2 min; room temperature for 5 min. (**f**) Bioresist was spin-coated with a 5 s slope; 500 rpm for 20 s; 5 s slope; 3000 rpm for 60 s; and slope for 10 s. (**g**) The bioresist was prebaked at 65 °C for 2 min, at 100 °C for 20 min, at 65 °C for 2 min, and at room temperate for 5 min. (**h**) The light intensity of UV exposure was 2000 mJ/cm^2^. (**i**) The bioresist was baked after the UV exposure at 65 °C for 2 min, at 120 °C for 20 min, at 65 °C for 2 min, and at room temperature for 5 min. (**j**) The substrate was developed in pure water at room temperature and was shaken at 60 rpm twice, 10 min each. (**k**) The substrate was then rinsed in pure water at 60 °C for 20 min. (**l**) N_2_ blow was performed to dry the substrate. (**m**) Finally, it was baked at 60 °C for 20 min.

Microstructure patterns were created on a glass substrate by standard photolithography (Fig. 2). 4-inch glass substrates (Toshin Riko, Japan) were cleansed using H_2_O_2_ and H_2_SO_4_, in 1:1 volume. The substrate was then kept at 100 °C for 15 min and then it was blown using an N_2_ blow. Bioresist-I and primer were obtained from Nissan Chemical (Japan) and returned to room temperature before spin-coating. The wafer was baked on a hot plate (EC-1200 N, AS ONE, Japan) at 180 °C for 10 min to remove water molecules on the surface. Then, it was allowed to cool at room temperature for 5 min. HDMS (Hexamethyldisilazane, OAP, Tokyo Ohka Kogyo, Japan) was spin-coated at 500 rpm for 20 s and 3000 rpm for 60 s over the wafer with a spin-coater (1H-DX-2, Mikasa, Japan). The wafer was baked at 65 °C for 2 min, at 120 °C for 10 min, and at 65 °C for 2 min. It was brought to room temperature and kept for 5 min.

Bioresist was spin-coated at 500 rpm for 20 s and 6000 rpm for 60 s over the wafer. The wafer was baked at 65 °C for 2 min, at 100 °C for 20 min, and at 65 °C for 2 min, at room temperature for 5 min. Hole patterns were exposed through a film mask using a mask aligner (Union Optical, PEM-800). The light integral to the bioresist was 2000 mJ/cm^2^. Bioresist is a negative photoresist, and the exposed areas polymerized and turned insoluble to water. The wafer was developed in water for 30 min two times and rinsed in hot water at 60 °C for 40 min. The wafer was finally baked on the hot plate at 60 °C for 20 min.

### Cell capture experiment

The developed bioresist pattern was cleaned and sterilized thoroughly before cell culture. The bioresist was rinsed with pure water and then dried with N_2_ blow. The substrate was treated in air plasma with a plasma asher (J-Science Lab, JPA-300) at 150 W for 200 s. It was then placed in a 2-inch petri dish and 10 mL of MEM (minimum essential medium) was added. 400 µL of HeLa cell (RCB0007, Riken BRC) suspension (density = 3.0 × 10^5^ cells/mL) was then seeded into the medium. The substrate was then cultured in the incubator (37 °C, 5% CO_2_) for 4 days. To inspect the cellular conditions, the substrate was taken from the incubator after 4 days and the cell medium was removed with an aspirator. PBS (phosphate-buffered saline, Gibco, USA) was added to wash the cells. The patterns with the seeded cells were finally observed under a microscope (ECLIPSE TE2000-U, Nikon, Japan) and a microscope camera (DS-Fi2, Nikon, Japan). Apart from the cell observation, we have also observed the nature of the developed bioresist pattern under normal incubation conditions (mentioned in the Results section). For observing the bioresist patterns without the cells, firstly, the bioresist was rinsed with pure water and dried with N2 blow. The substrate was then plasma treated with a plasma aching device (J-Science Lab, JPA-300) for 150 W for 200 s and put in a petri dish filled with 10 mL MEM (in 1% FBS and PS, Gibco). The resultant dish was then put into an incubator maintained at 37 °C and in 5% CO_2_ (MCO-5AC, Panasonic, Japan). Initially, we observed the behavior of the bioresist patterns after 3 days of continuous incubation.

### Data preparation

Learning experiments were performed on two different collected images. Bright-field images and fluorescent images were captured with the microscope camera. All the collected images were pre-processed and augmented. Image augmentations are necessary because they introduce additional noise to the image that helps the model understand robust information from a particular image. The image augmentations that have been preferred for experiments here are random flips, random rotations, and mirroring. Each of the augmentations caters to different functions in an image. When applied to an image, random flips flip the image by a random degree. Random rotations rotate the image on the z-axis by a random degree. Mirroring an image simply mirrors the image. Since the image contains information on the location of the bioresist patterns and whether the patterns contain cells or not.

The images were labeled manually using LabelImg, a freely distributed labeling software available in Python. The labels were created according to the PascalVOC data format, thus storing the label information in XML. The dataset was designed to have two classes: ‘cells’ and ‘no cells’. The class ‘cells’ suggests the bioresist pattern has cells in them. Whereas, the class ‘no cells’ suggests that the bioresist pattern doesn’t have any cells in them. The initial dataset was prepared using bright-field images only.

### Evaluation metrics

Some important metrics of each model’s functioning are considered for finding the optimal model for the cell detection task. The mAP (mean average precision) value defines the ability of the model to understand desirable features from images. We have mostly considered models that provided an mAP value greater than or equal to 0.75. Mean Inference time is considered because the model has been theorized to achieve high detection performance in near real-time. Loss values (Classification loss, box plot loss, total loss, etc.) are used in this study to check whether the model is reaching an over-fitting condition or not. They didn’t directly contribute to the learning and final model testing process.

### Deep learning models

In this study, we have explored three state-of-the-art deep learning models. These models were selected because they fit our initial criteria of having an mAP value greater than 0.75. The models that have been explored here are namely, RetinaNet, RPN (Region Proposal Network), and Faster RCNN.

#### RetinaNet

The RetinaNet^25^ is a state-of-the-art single-stage detector that introduces the novel focal loss method for dealing with the extreme foreground–background class imbalance problem, by reshaping the standard cross-entropy loss. RetinaNet follows the Pyramid structure of convolutional networks for getting semantic information from images. This method employs proportionately scaled feature maps at various levels from a single-scale picture of any arbitrary size. ResNet^26^ has been preferred for this study because of its skip connections thus retaining previously learned information.

#### Region proposal network (RPN).

Convolutional feature maps from the most recent shared layer are used to produce region predictions by sliding a small network across them. At each sliding-window location, the base algorithm^27^ predicts multiple region proposals, and the maximum number of proposals is controlled by anchors, denoted by *k*. The anchors used in Faster RCNN are translation-invariant, meaning that when one translates an object into an image, the proposals re-translate them according to the new object, and no other external functions are required for this task. This feature has indeed reduced the overall number of parameters in the network. For training the RPN, a binary class label was assigned to each anchor. All anchors with an Intersection over Union (IoU) value higher than 0.7 are labeled as positive anchors and the rest as negative.

#### Faster RCNN

The faster RCNN^27^ module is a two-stage detector where the first stage is used to propose regions of interest and based on those regions the Fast RCNN detector predicts the classification label. The faster RCNN module has implemented an attention network thus simplifying the module to look at specific regions of interest. The proposals of the regions of interest are done by the RPN network as mentioned above. Faster RCNN has been designed to have an alternate training method where the RPN generates the proposals over the convolutional feature maps and then based on the proposals the classification detector (Fast RCNN) generates the classification label. The networks are initialized using the ImageNet dataset^28^ separately. Next, the initial layers were fixed, and these fixed layers were shared with the RPN network for region proposals. After fixing, only the layers that are exclusive to the networks are changed during training and the rest remain fixed, thus creating a unified network.

### Color space Information

For testing the understanding of the model in different color spaces, we experimented with the developed model under different color spaces. Primarily, the model was trained using RGB images. Later, we used different color spaces, including YUV (Brightness, blue projection, red projection), HSV (Hue, Saturation, Value), and CIELAB (Commission Internationale de l´Eclairage color format). The YUV color space has been historically used for a specific analog encoding of color information in a television system. When represented in the form of RGB, the YUV uses different forms of illumination ratios of RGB to display its properties.

$$ \left[ \begin{gathered} Y \hfill \\ U \hfill \\ V \hfill \\ \end{gathered} \right]\, = \,\left[ \begin{gathered} 0.299\,\,\,\,\,\,\,\,\,0.587\,\,\,\,\,\,0.114 \hfill \\ - 0.147\,\, - 0.288\,\,\,\,\,\,0.436 \hfill \\ 0.615\,\,\,\,\,\, - 0.515\,\, - 0.1 \hfill \\ \end{gathered} \right]\left[ \begin{gathered} R \hfill \\ G \hfill \\ B \hfill \\ \end{gathered} \right] $$

Similarly, for CIELAB, a specific mathematical form of the RGB color values is utilized such that we get a latent uniform representation when the computer starts reading the information from the images. However, for converting the initial RGB images into CIELAB, firstly the images are required to be converted into XYZ format and then subsequently transformed into CIELAB. A similar approach has been taken while converting from RGB to HSV. Moreover, HSV deals with the Hue, Saturation, and Value of the color information already present in the image. In this study, we have utilized the OpenCV library of python for converting into all the desired color spaces. During training, to maintain a fair share of knowledge for all the color spaces, we didn’t pre-train the models for the different color spaces.

### Computational setup

**Figure 3:**
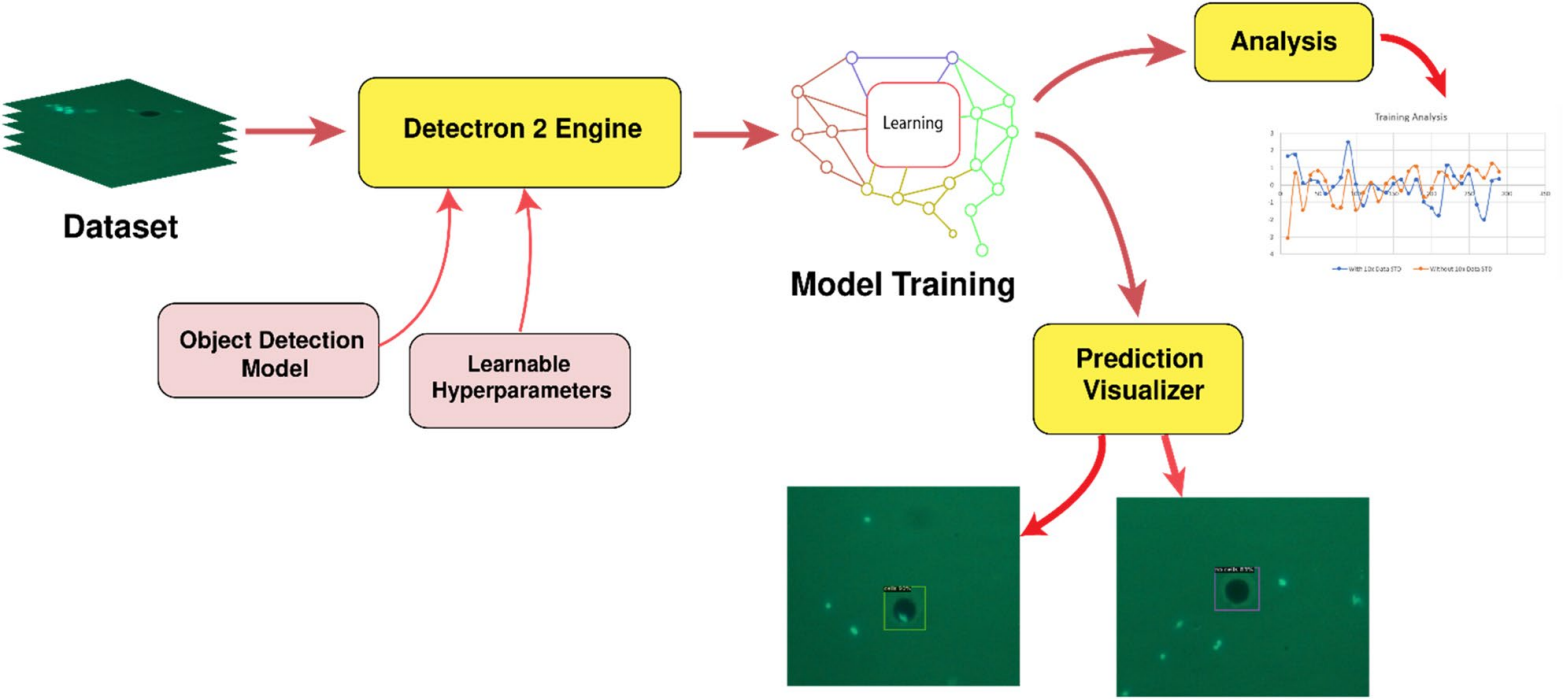
The overall workflow of the cell detection setup based on Detectron2. The analysis section doesn’t necessarily come as a part of the pipeline and is used to test the efficiency of the model and interpret the generated results. Visualizing predictions is preferred for any real-life implementations.

We trained the models for 3000 epochs and the initial learning rate was kept at 8 × 10^–4^. After a hit and trial run over the different datasets, the learning rate was decided to be 5.5 × 10^–4^. All the models were trained locally, using NVIDIA GeForce RTX 3070 GPU. Because of the restrictions of computation power that the local system offered, all the images were resized to 416 × 416 pixels. We have used 16 images per batch. We didn’t employ any weight decay for the models during the learning process. We optimized the network using binary cross-entropy for the classification of features task and mean squared error for the object detection and bounding box prediction tasks. We have adopted Detectron2 as an engine for training models on the cell detection dataset for ease of implementation (Fig. 3). As required by Detectron2, we have registered our dataset into the system of the engine, where the labels contained information about the bounding boxes and their respective classes. The bounding box information was stored as the minimum and maximum x and y coordinates respectively. The Detectron2 module also contained in-built visualization functions that facilitated the testing and visualization.

## Results

### Pattern diameter of bioresist

**Figure 4:**
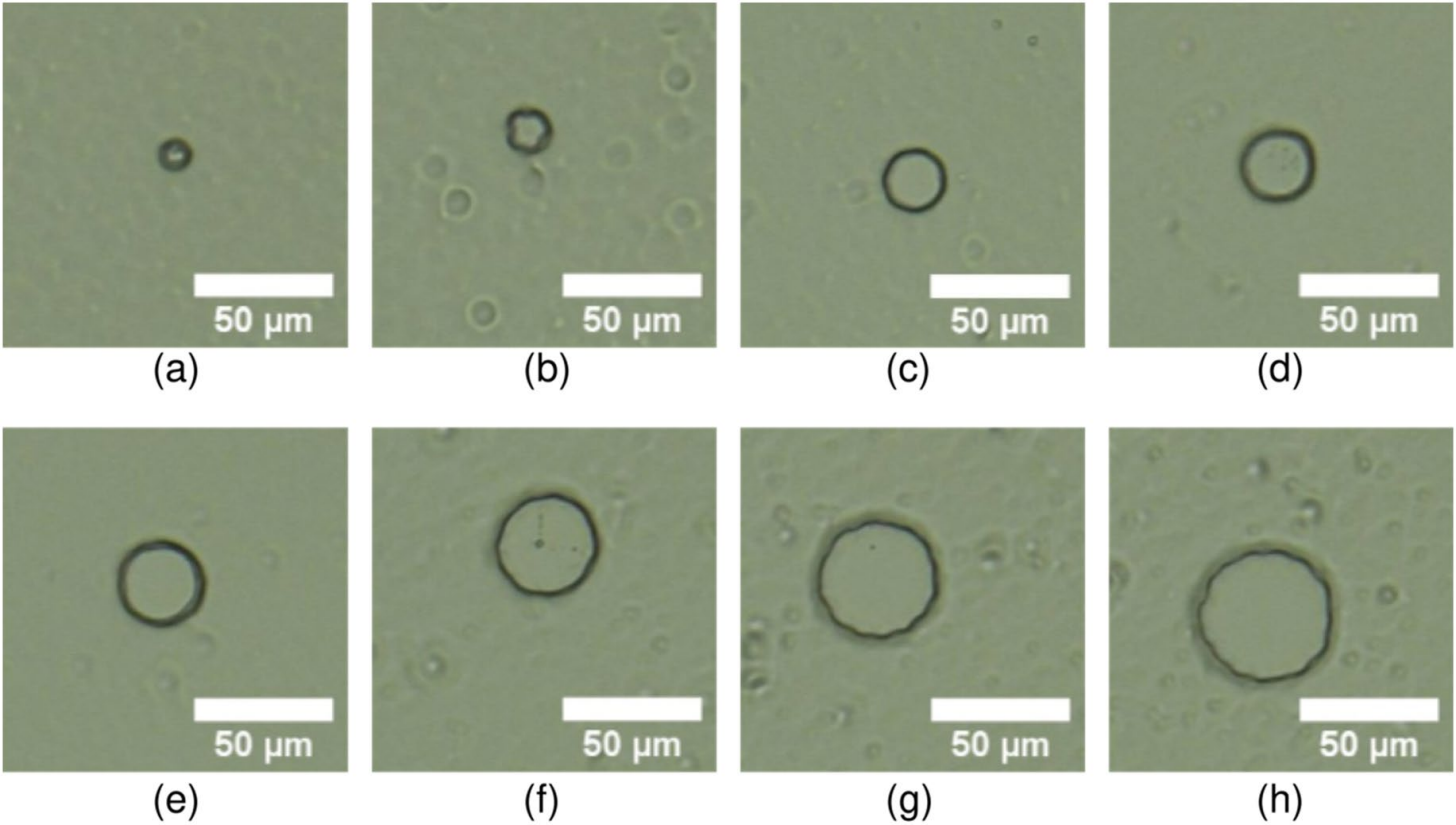
The concave structures of the bioresist patterns with different diameters. Pattern diameter (**a**) 10 µm, 15 µm, (**c**) 20 µm, (**d**) 25 µm, (**e**) 30 µm, (**f**) 35 µm, (**g**) 40 µm, and (**h**) 45 µm. The outside of the circles is coated with a bioresist.

We initially chose a wide range of diameters for the bioresist pattern. Seven successful substrates were prepared from single photolithography that was able to form the concave structures required for cell trapping. Figure 4 demonstrates the different bioresist patterns that we have developed for this study. Since our main aim was to develop long-term experiment methods, we required the patterned bioresist to satisfy those conditions. The developed bioresist patterns were tested under incubation for different time intervals, for simulating the cell environment. The residual film remained in some of the bioresist patterns. A possible way of reducing the residual film is to increase development time, increase the shear force on the bioresist, and increase the swing rotational speed during development.

**Figure 5:**
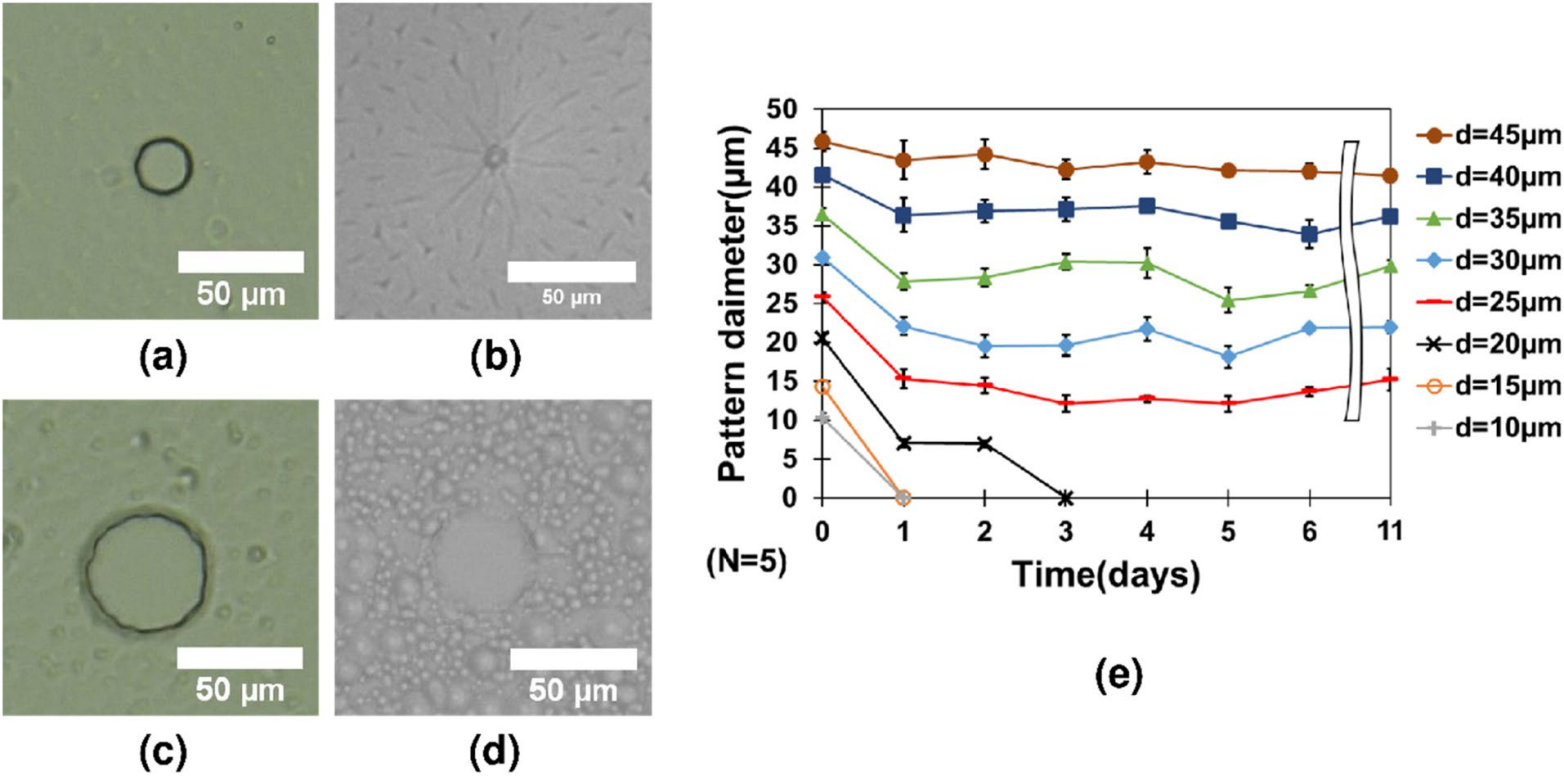
Behavior of microwell patterns under incubation for different timeframes. (**a**, **b**) The bioresist concave structure (d = 20 µm) was observed after 3 days of incubation. (**c**, **d**) The bioresist concave structure (d = 45 µm) was observed after 11 days of incubation. (**e**) The behavior of different bioresist patterns at different times of incubation. (**f**) The single-cell trapping efficiency was observed for different microwell diameter sizes.

Figure 5a,b show that the 20 µm diameter bioresist pattern disappeared after 3 days of incubation. The concave structure closed mainly because of absorbing moisture. Similar behavior was observed in other patterns as well. At 25 µm, the hole diameter was reduced to around 15 µm after 1 day. Empirically, it was not possible to run experiments for a longer period because of the considerable reduction in the diameter size of the bioresist patterns. Similarly, the microwell of pattern diameter of 45 µm was able to stay intact after 11 days of incubation (Fig. 5c,d). This is suggestive of the fact that bioresist patterns with higher diameters are necessary for our experiments on higher incubation times.

Figure 5e reflects the behavior of the microwell patterns during different time intervals. All the microwell patterns below 20 µm diameter size disappeared after 3 days of incubation. However, as we have enlarged the size of the microwell patterns, they have sustained longer days of incubation time.

### Cell trapping and Viability

**Figure 6:**
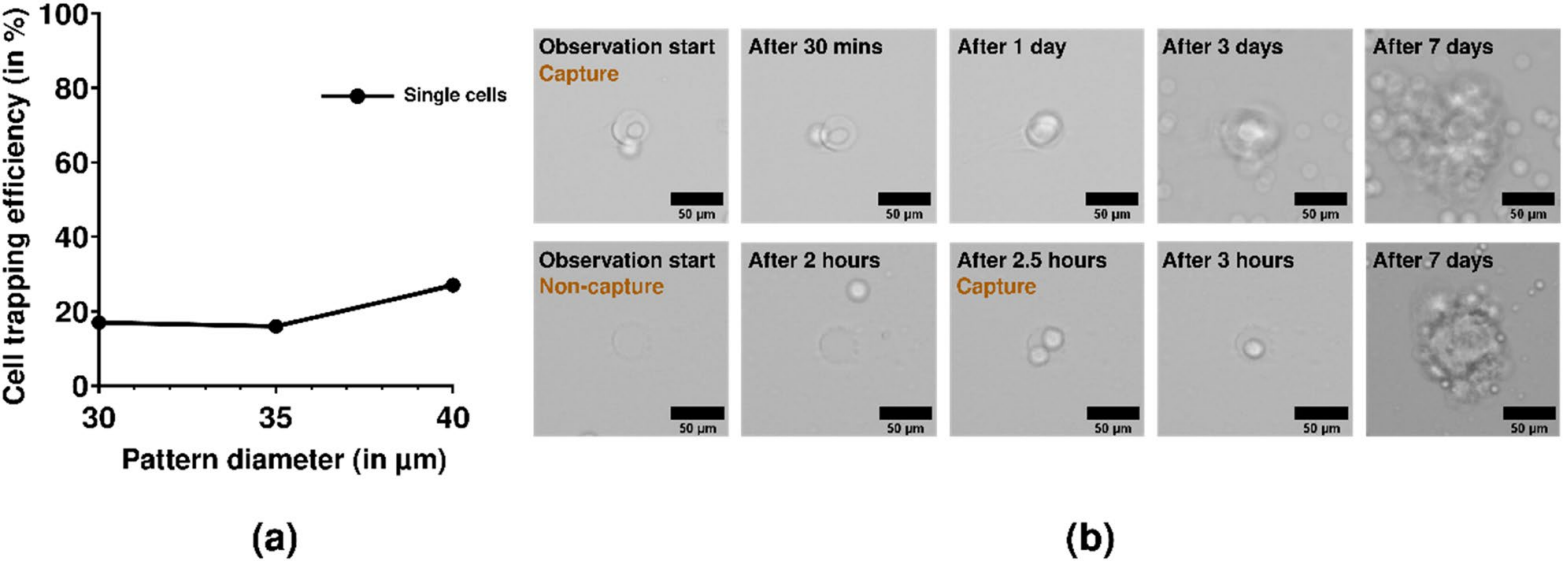
Cell trapping efficiency and the growth of a trapped single cell. (**a**) The single-cell trapping behavior observed against the diameter size of the microwell array. (**b**) Cell viability was observed for a period of 7 days. After 7 days of incubation inside the microwell, cells divided in both instances.

It is interesting to note that with the increase in the diameter size, there has been an increase in the cell trapping phenomenon. Figure 6a reflects the behavior of the single-cell trapping efficiency of the method for different microwell diameter sizes. Cell trapping efficiency was 43% at 45 µm, but two or more cells were trapped at this microwell size. Pattern diameters from 10 to 25 µm didn’t capture any cells. Hence, we have chosen to perform experiments for microwell diameters of 30 to 40 µm, where we majorly observed trapped single cells. Figure 6b shows cell division for 7 days after cell capture confirming the biocompatibility of our method.

### Dataset preparation

**Figure 7:**
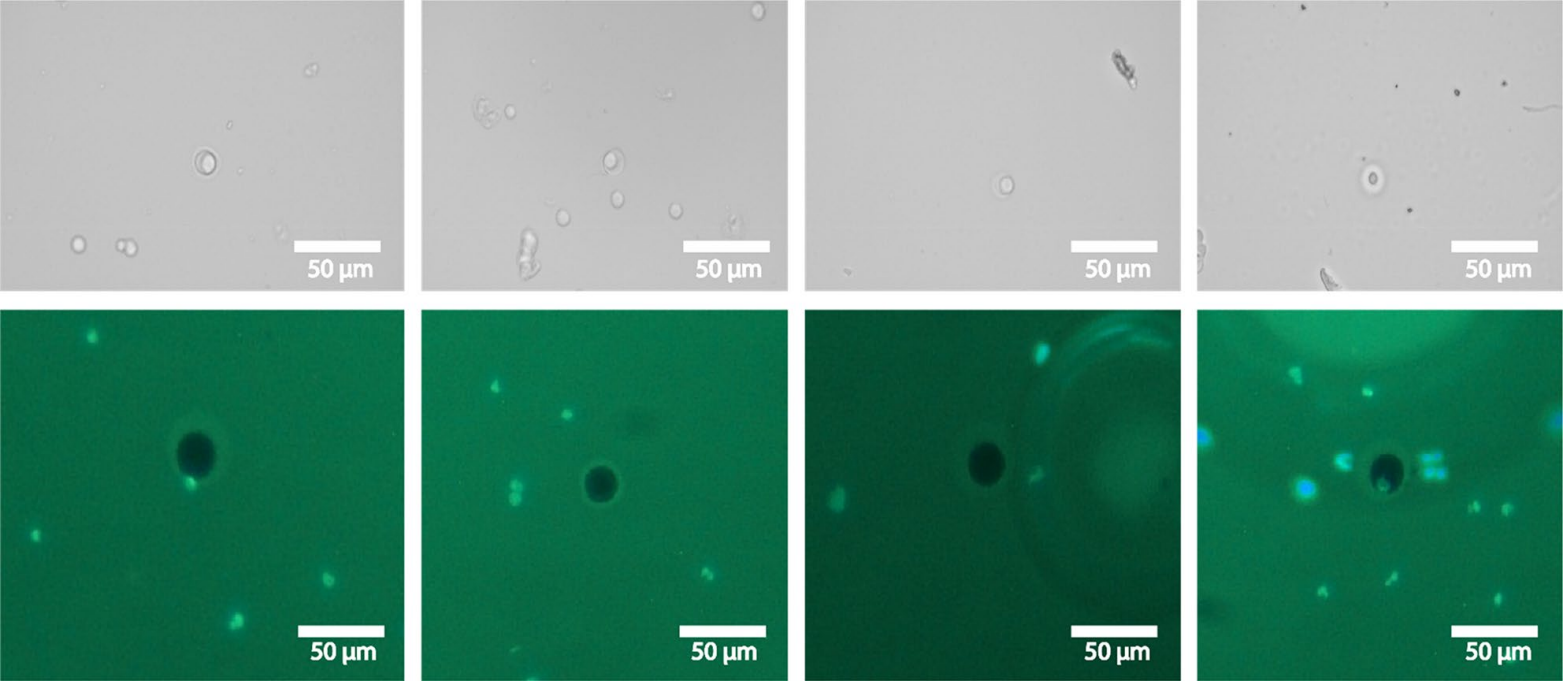
Images in the bright-field microscopy and fluorescence microscopy datasets. It turned out that there was an issue with the contrast because of which the artifacts were not observable. Later, we resorted to fluorescence microscopy images (bottom), where each artifact was distinctive and observable.

**Figure 8:**
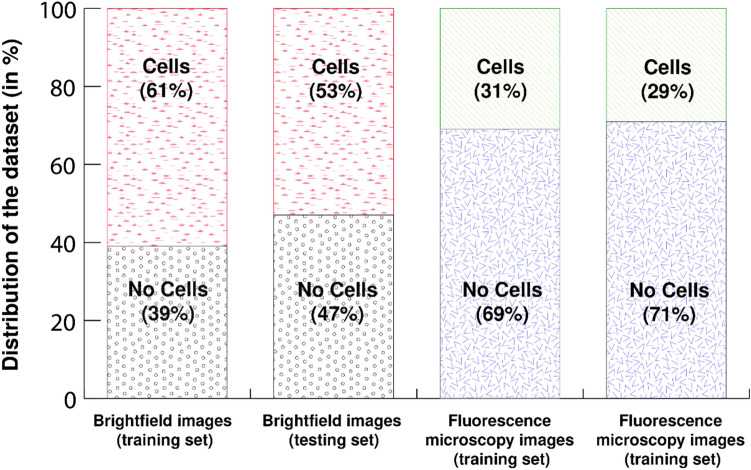
The distribution of images in either of the datasets, according to the presence of cells in the microwells. Our datasets had a greater number of images in which there were cells trapped. However, in the latter part of the experiments, when we chose fluorescence microscopy images, we obtained images of microwell patterns where no cell trapping was observed for a majority section of the dataset, for both the training and testing set. This in turn has ensured that our model sufficiently learns from those images as well where the cells are not trapped in the microwells.

We have considered the microwell array of size 30 µm for our further developments. The bright-field images taken (Fig. 7 top) were classified into two distinct sections: the training dataset and the testing dataset. The training dataset had 322 bright-field images. Out of them, 197 contained cells and 125 didn’t contain cells in the bioresist pattern (Fig. 8). The testing dataset, on the other hand, had 107 images. Out of them, 57 contained cells and 50 didn’t contain cells in the bioresist patterns. It was confirmed manually that no duplicate images are present in either of the datasets.

The fluorescent images were taken in bulk and then were later manually cropped into individual bioresist patterns to facilitate the detection process. A similar process has been followed to create two distinct datasets for model training and testing (Fig. 7 bottom). 1035 images were prepared as a training dataset composed of 321 cell-contained images and 714 no-cell images in the bioresist pattern (Fig. 8). The testing dataset contained 34 images, 13 contained cells, and 21 didn’t contain cells in the bioresist patterns. In this study, we have studied both datasets to find the optimal parameters for cell detection in such bioresist patterns.

The fundamental difference between the different datasets was found in Fig. 7. At a glance, the brightfield images had a contrast issue because of which there was not much observable difference between our region of interest and the background. On the other hand, fluorescence microscopy images have visible differences between the region of interest and the background, thus making it quite easier for the models to extract latent information from the images and learn the features efficiently.

### Automating single-cell detection

All the models were trained locally. We conducted experiments with the three mentioned object detection models on both of the created datasets. All the results mentioned in bold indicate the best-achieved results.

In Table 1, RetinaNet performs the best in terms of mAP and average inference time. The mAP for RetinaNet has reached 80.13%, with an average inference time of 0.09 s. However, it didn’t perform well in terms of the total losses received after optimization of the respective loss functions. The least losses were achieved by RPN. Their architectural design explains this behavior of the RPN and Faster RCNN having better loss values than RetinaNet. Both RPN and Faster RCNN, being two-stage detectors, share some convolutional layers among their region proposal networks and classification networks. This keeps their main network shallow and hence since knowledge is pre-defined (from the shared convolutional layers), the loss for shallow models also becomes lesser. But as the RetinaNet, is a single-stage detector, both the detection and classification tasks take place at the same time with no shared convolutional layers in between, thus making the model have higher losses than the rest.

**Table 1:** Comparison of mean average precision, losses, and average inference time of different models on the fluorescence image dataset.

Model	mAP (Mean Average Precision)	Total loss	Classification loss	Average inference time (s)
RetinaNet	**0.8013**	0.1634	0.023	**0.09**
RPN	0.7355	**0.033**	**0.012**	0.10
Faster RCNN	0.6475	0.0615	0.0132	0.12

**Figure 9:**
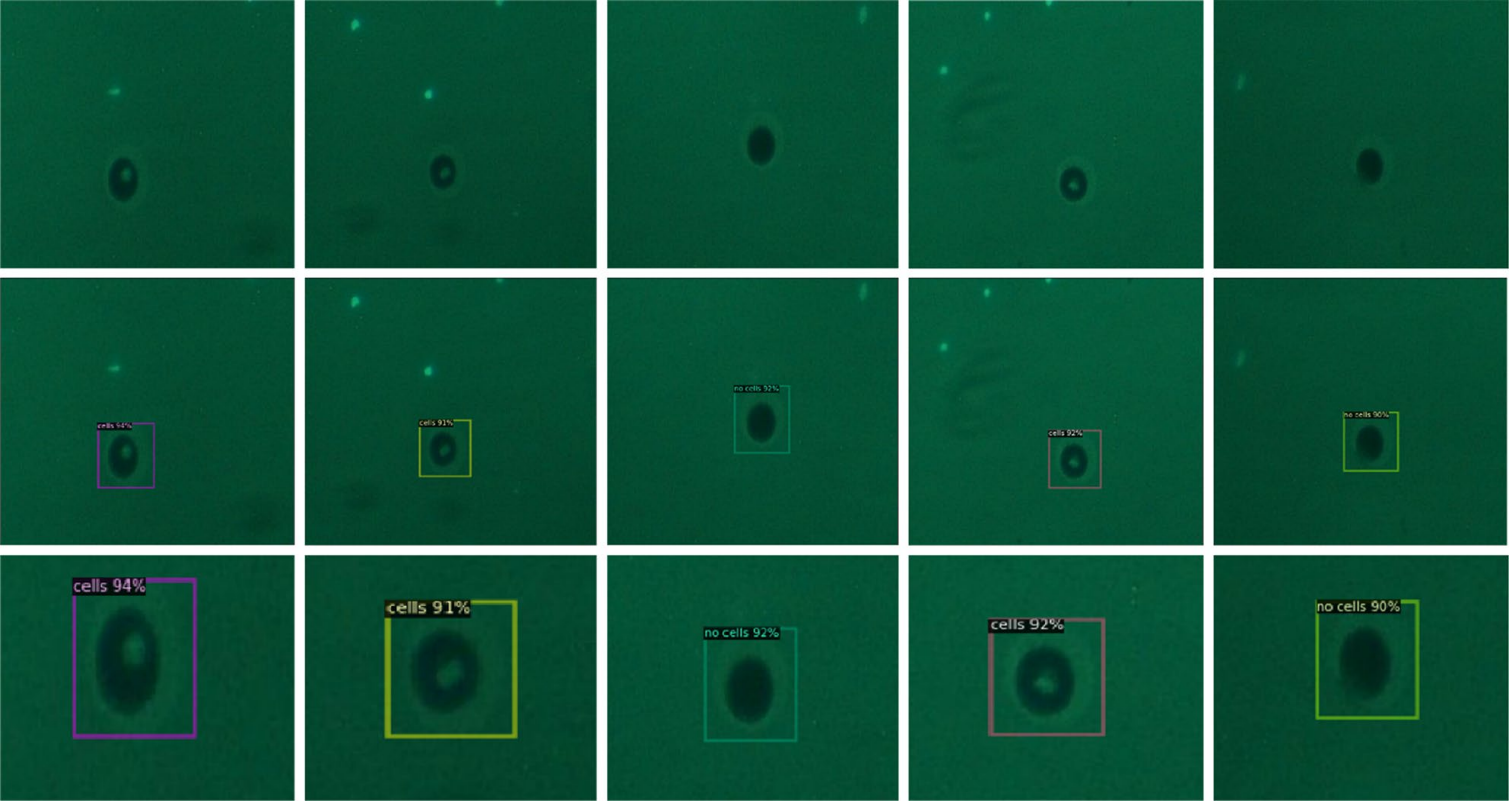
The detection obtained from RetinaNet. We have considered an ROI (Region of Interest) threshold of 0.85 and tested the results via CPU for visualization. This also ensures the compatibility of the method in resource-limited environments. (Top) The original images were put into the DL model for testing. (Middle) The results depict the region of interest and the location of the microwell. In addition to that, the model is classifying the images based on whether the microwell patterns contain cells or not. (Bottom) An enlarged sectional view of the detected microwells. The last row of images clearly shows the labels associated with each of the microwells, as detected in the *middle*-row images.

Table 2 represents the performance values of different models using the fluorescent image dataset with some drastic changes. With the addition of color channels in the images, the detection accuracy has increased drastically. Similar trends are observed in Table 2 as well. RetinaNet performed the best with 98.9% mAP values and an average inference time lower than the corresponding bright-field image dataset results. Similarly, total losses were observed to be lowered for RPN and Faster RCNN as well. The addition of color information to cellular images, as we have used in our dataset, increases the overall detection accuracy, and decreases the average inference time. The predictions from RetinaNet could be observed in Fig. 9. Some cells were observed in the vicinity of the microwell. Those cells won’t affect the downstream cell analysis or detection, because this model was trained to look for the microwell structures and then locate single cells in the microwell bounding boxes.

**Table 2:** Comparison of mean average precision, losses, and average inference time of different models on the fluorescence image dataset.

Model	mAP (Mean Average Precision)	Total loss	Classification loss	Average inference time (s)
RetinaNet	**0.989**	0.2048	0.040	**0.06**
RPN	0.7143	**0.019**	**0.012**	0.072
Faster RCNN	0.9557	0.0704	0.0163	0.093

### The color information in the detection

From the detection results in brightfield images and fluorescent images, adding color information to the images increases the detection accuracy by about 53%. We explored the importance of color information in the images during the training session.

**Figure 10:**
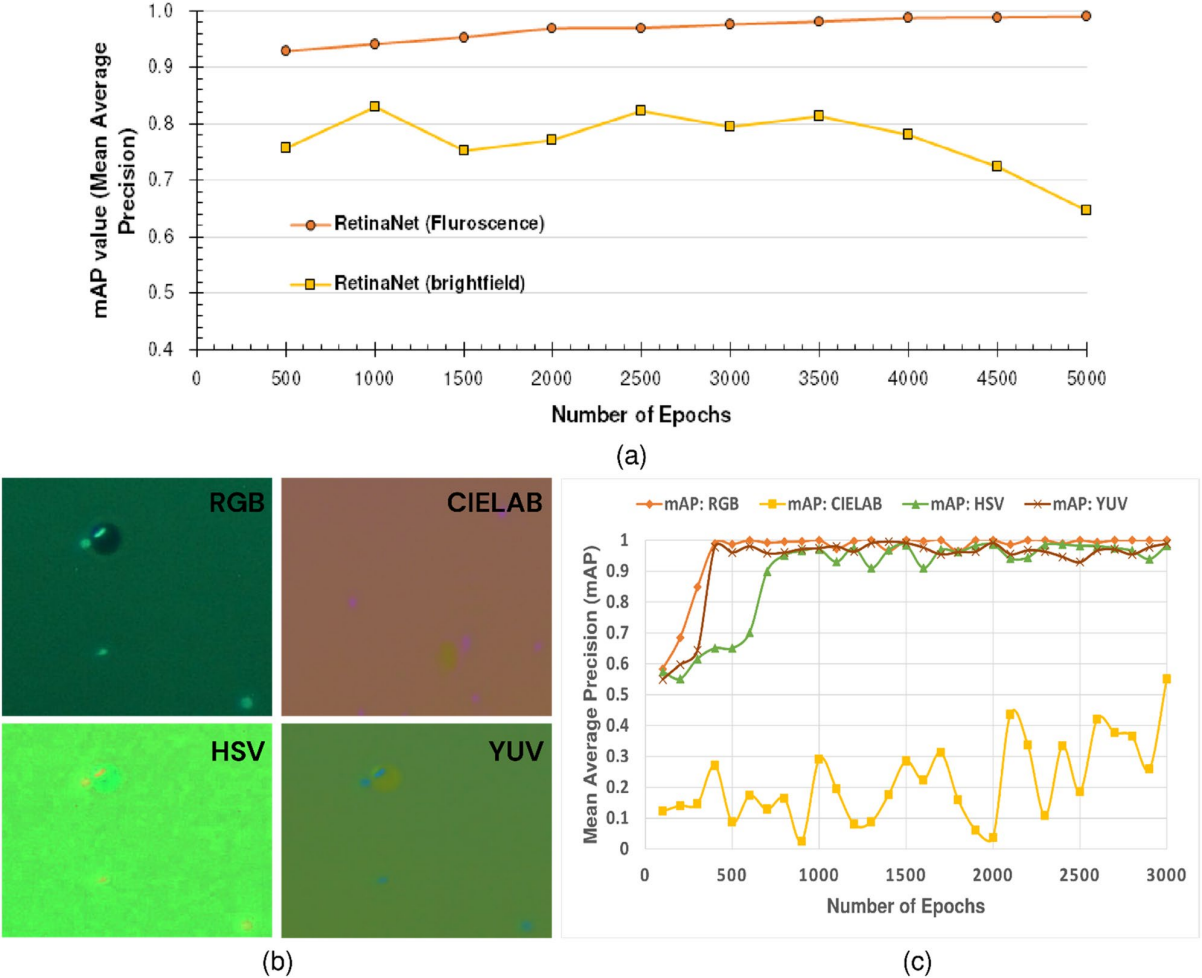
Training mAP of RetinaNet in brightfield and fluorescent image datasets. Brightfield images didn’t contain any color information whereas fluorescent images had color information. (**a**) The training was done for 5000 epochs initially for both the brightfield images and fluorescent images. (**b**) The training was re-done on images of different color spaces. (Top to bottom) The different color spaces include RGB, CIELAB, HSV, and YUV, respectively. (**c**) The same model was re-trained on the above-mentioned datasets and portrays the importance of color as a feature for detecting artifacts in our microscopic images.

In Fig. 10, only RetinaNet has been tested as a reference to show the influence of colors in the detection process. After 500 epochs of training, the same model achieved more than 90% accuracy for the fluorescent image dataset and achieved above 70% accuracy for the brightfield image dataset. Further in the training process, there have been overall fluctuations in the learning phase for brightfield images showing the inability to converge from the learned knowledge. Also, when trained with brightfield images, the model was not able to outperform the model trained in fluorescent images. Additionally, after 3500 epochs of training, the model is not able to learn anything, and the accuracy of detection is gradually decreasing. This phenomenon suggests that the model is overtraining itself, thus learning too much from the training dataset and not being able to generalize in the validation/testing dataset.

To confirm our hypothesis that image color is an important aspect in the detection of microwells and cells present in them, we have tested the same model in the same dataset but with different color spaces. For this experiment, we have compared 4 different color spaces, including RGB, CIELAB, HSV, and YUV. Each of these color spaces contains different information about the image.

CIELAB was facing the highest errors with the least accuracy in the detection for our case because of the undetectable artifacts in the images. However, for RGB and YUV, we see similar results because of noticeable and distinctive characteristics. For HSV, we notice that the learning was similar compared to RGB and YUV images, but the model initially faced difficulty in noticing some of the important features in the dataset. However, in the later stages of training, it was easily able to converge.

## Discussions

An experimental approach of single-cell trapping has been introduced in this study, for single-cell extraction. Moreover, this study introduces a novel method of cell patterning using hydrogels. Here, we have used a bioresist to pattern microwell arrays and used them for single-cell trapping. We have experimented with different dimensions of the microwell patterns and have empirically shown the desired dimensions fitted according to the needs of the cell trapping experiments. In addition to that, we have mentioned the pattern shrinking phenomenon, when kept under incubation for longer periods. This phenomenon has shaped our final design considerations. Our study on using bioresists for cell patterning is rather novel as it is biocompatible, in addition to that, it maintains the high-throughput efficiency of the experiments, even for longer incubation time.

Moreover, since checking every microwell and manually determining a cell trapping phenomenon is an inefficient approach, we have automated the system using machine learning where the implemented system efficiently detected the microwell patterns and classified whether the patterns contain cells or not. To the best of our knowledge, this is the first study that mentions detecting the single cells trapped in novel patterned bioresists in near real-time. We have implemented our system using Detectron2 because of its extensive functionality that not only helps in training but also helps in validation analysis. Initially, we had chosen bright-field microscopy images but essentially all our models didn’t perform well as compared to the models when we changed to fluorescence microscopy images. For RetinaNet, we were able to converge at an mAP of 0.989 and around 1200 epochs. Upon comparing with the existing literature, we found that our development was empirically performing better, in terms of mAP and accuracy. Similar models were deployed for cancer cell counting and detection in zebrafish xenografts^29^. The study mentioned that the entirely fine-tuned model was able to achieve an mAP of 0.8457, considering all the background noise in the images and avoiding all the unwanted small artifacts in the images. Earlier, Faster RCNN was implemented for automated blood cell detection and counting^30^. The authors have mentioned achieving a detection accuracy of 0.984, which is comparatively very much close to our results. Another implementation of cell counting was done where the authors tried to improve the blood cell component counting by using different versions of YOLO. Cell counting is generally preferred in blood cells as they tend to reveal information regarding the growth of any cancerous cells. The results mentioned having achieved an mAP value of 0.943^31^. Hence, our developed model for this specific application outperformed all similar tasks, related to cell object detection under different environments. Our developed model is limited based on the dataset obtained with a microwell array of size 30 µm, which trapped mainly single cells. The images of multiple cells trapped at this level were low in number compared to the entire dataset. Adding such a low number of images, compared to the prepared dataset caused skewness in the dataset. The model would become biased and disregard such images, leading to errors in prediction.

Moreover, during the training of both of our datasets, we noticed an anomaly regarding the number of channels in the images of our dataset, which essentially determines the color information in an image. This gave an initial hypothesis that color images might be important for our model to function properly and hence to confirm that hypothesis, we changed the color spaces of our dataset and performed validation. We found that for RGB and YUV images, the results were almost similar, and the model was able to detect the artifacts of the images at fewer iterations. However, when we turned to CIELAB images, we found that because of the obvious unclear features of the images, the model failed to converge, and even after 5000 epochs of training, the model failed to detect the desired features.

## Conclusions

In this study, we have demonstrated the application of bioresist to trap single cells in microwell arrays. The microwell patterns were prepared over a glass substrate using a standard photolithography process and the behavior of the developed patterns was explored under different incubation times. The advantage of our method is that it is biocompatible, without the use of harsh chemicals. This suggests longer higher cell viability. Moreover, our method is capable of withstanding higher incubation times and microwell arrays above the size of 30 µm retained their shape, structure, and properties, even after an incubation time of 11 days. Our method is easy to apply because of a standard photolithography process, which makes it easy to start the process. In addition to that, we have introduced a DL system that efficiently detected and located the microwell patterns and classified the presence of trapped cells in the same. We were able to optimize the model such that it was achieving an mAP of 0.989 and at an inference time of 0.06 s, making the entire system fast and reliable.

## Data Availability

The dataset for the object detection model, consisting of the microwell patterns with cells seeded in them, has been uploaded to Google Drive: https://drive.google.com/file/d/1g4I85wAyJ-j-70el6vtU9lca14s2Fl7W/view?usp=sharing. The dataset has been made accessible to all. The uploaded dataset contains all the brightfield image and fluorescence image data used in this study, with the corresponding labels of each image stored in XML files.
